# HPV-16 E7-Specific Cellular Immune Response in Women With Cervical Intraepithelial Lesion Contributes to Viral Clearance: A Cross-Sectional and Longitudinal Clinical Study

**DOI:** 10.3389/fimmu.2021.768144

**Published:** 2022-01-13

**Authors:** Lina Zhang, Xinyi Shi, Qing Zhang, Zhilei Mao, Xiaoyu Shi, Jun Zhou, Aili Jian, Renying Zhu, Shisong Jiang, Wenshu Lu

**Affiliations:** ^1^ Center for Diagnosis and Treatment of Cervical Diseases, Changzhou Maternal and Child Health Care Hospital, Nanjing Medical University, Changzhou, China; ^2^ R & D Department, Oxford Vacmedix (Changzhou) Co. Ltd., Changzhou, China; ^3^ Department of Microbiology and Immunology, School of Basic Medicine, Dali University, Dali, China; ^4^ Department of Oncology, University of Oxford, Oxford, United Kingdom; ^5^ R & D Department, Shanghai Jia Wen (JW) Inflinhix Co. Ltd., Shanghai, China

**Keywords:** recombinant overlapping peptide, human papillomavirus, cervical intraepithelial neoplasia, immune responses, cervical cancer

## Abstract

High-risk human papillomavirus (HPV) infection is the cause of almost all cervical cancers. HPV16 is one of the main risk subtypes. Although screening programs have greatly reduced the prevalence of cervical cancer in developed countries, current diagnostic tests cannot predict if mild lesions may progress into invasive lesions or not. In the current cross-sectional and longitudinal clinical study, we found that the HPV16 E7-specific T cell response in peripheral blood mononuclear cells of HPV16-infected patients is related to HPV16 clearance. It contributes to protecting the squamous interaepithelial lesion (SIL) from further malignant development. Of the HPV16 infected women enrolled (n = 131), 42 had neither intraepithelial lesion nor malignancy (NILM), 33 had low-grade SIL, 39 had high-grade SIL, and 17 had cervical cancer. Only one of 17 (5.9%) cancer patients had a positive HPV16 E7-specific T cell response, dramatically lower than the groups of precancer patients. After one year of follow-up, most women (28/33, 84.8%) with persistent HPV infection did not exhibit a HPV16 E7-specific T cell response. Furthermore, 3 malignantly progressed women, one progressed to high-grade SIL and two progressed to low-grade SIL, were negative to the HPV16 E7-specific T cell response. None of the patients with a positive HPV16 E7-specific T cell response progressed to further deterioration. Our observation suggests that HPV16 E7-specific T cell immunity is significant in viral clearance and contributes in protection against progression to malignancy.

## Introduction

Cervical cancer is a malignant tumor that seriously threatens the life and health of women. Globally, approximately 500,000 women are diagnosed with cervical cancer every year and of these 280,000 women die from cervical cancer (1). It is an accepted fact that high-risk human papillomavirus (HR-HPV) infection is the main risk factor for the occurrence, persistence, and development of cervical cancer (2). For disease control, hybrid capture 2 (HC-2) or HPV-DNA typing combined with cytology examination are the diagnostic methods used to detect cervical cancer (3). However, these methods cannot distinguish invasive lesions from noninvasive lesions, nor do they provide any prognostic value for patients. In fact, only about 1% of women infected with HR-HPV will gradually develop cervical cancer (4, 5). HPV infection in most women is only temporary or does not lead to cervical cancer even if HPV persists. Currently once HPV-infected lesions have been diagnosed, patients are often over treated with unnecessary medical procedures including surgical interventions (1). To avoid over treatment, it is important to understand the mechanisms of viral clearance followed by developing practical methods to monitor the process. If the viral clearance mechanism exists, it may not be urgent for HPV-infected women to undergo surgery.

HPV-related carcinogenesis is not a uniform process. The “integration” of the virus into the host cell increases gene instability, marking the beginning of malignant transformation (2). It takes approximately 10 years from HR-HPV infection to develop cervical carcinogenesis (6). HPV infection triggers a series of immune responses, namely, innate and adaptive immune responses. Consequently, the occurrence of cervical cancer is the result of the struggle between foreign HPV infection and self-immunological defense (4). In most circumstances, the body’s own immune function will resolve the infection, prevent further viral invasion and pathogenesis, eliminate damaged or aging cells, and address abnormal transformed cells in the body. However, when the immune surveillance function of the body is compromised or dysfunctional, it will result in a decrease in the ability to eradicate abnormal transformed cells. This will lead to high-level lesions and perhaps the occurrence of a malignant tumor (4, 7).

Among all high-risk HPVs, more than half of cervical cancer patients around the world are infected with HPV16 (8). The responses of cytotoxic lymphocytes (CTL) to E6/E7 appeared to be important in the prevention of squamous interaepithelial lesions (SILs) (9, 10). Strict conservation of HPV16 E7 is critical for HPV16 carcinogenesis (11). Therefore, effectively detecting an HPV16 E7-specific immune response may act as an immune control mechanism to prevent persistent HPV infection and to predict the progress of the disease. Thus, immune function, especially T cell-based immunity, is at least one of the key factors that are relevant to the prognosis of HPV infection and its malignancy. However, there is no commercially available test for monitoring T cell immunity.

In this study, we used the recombinant overlapping peptide protein of HPV16 E7 (ROP-HPV16 E7) and the E7 overlapping polypeptides as stimulants in the Enzyme-linked immunospot (ELISPOT) assay to evaluate the HPV16E7 specific-T lymphocyte response in peripheral blood mononuclear cells (PBMCs) of patients. The results showed that the low HPV16 specific T cell response in peripheral blood was significantly correlated with persistent viral infection. Our study suggests that the HPV16-specific T cell response in peripheral blood can effectively predict clinical outcome.

## Results

### Effective Detection of HPV16-Specific T Lymphocytes in the Peripheral Blood

**Figure 1:**
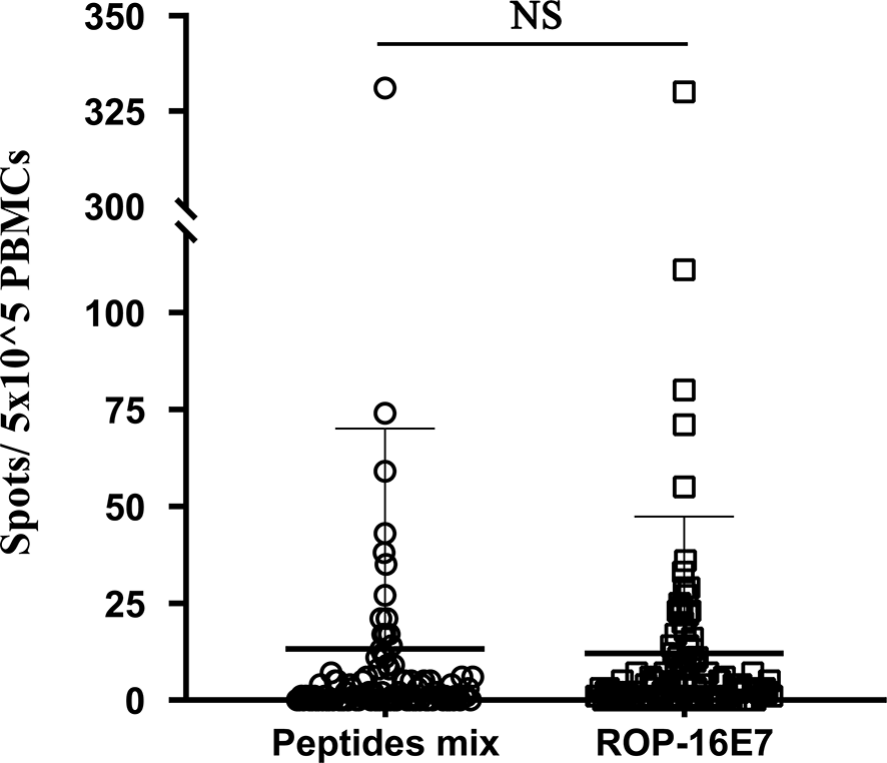
IFN-γ release assay of patient-derived PBMC cultures that recognize HPV16-specific peptide pools (peptides mix) and ROP-16 E7. NS, no significance.

In our previous study, ROP-HPV16E7 effectively stimulated the HPV16 E7-specific T cell response in mice immunized with the HPV protein (12). In this study, using patient PBMCs infected with HPV16, we compared the ability of ROP-16 E7 with a pool of HPV16 E7 overlapping peptides to simulate the specific T cell response against HPV16 E7. ROP-16E7 stimulation mimicked that of pooled HPV16 E7 peptides to effectively stimulate HPV16 E7-specific T cells in patients infected with HPV16 (**Figure 1**). No significant difference was found between ROP-16 E7 and HPV16 E7 pooled peptides. Therefore, ROP-16E7, a recombinant protein that can be easily and abundantly expressed and purified from *E. coli*, is able to replace the costly, and difficult to manufacture, quality-controlled pool of HPV16 E7 peptides, in the interferon-γ release assay (IGRA).

### Recruitment of Patients and Their HPV16 E7-Specific T Lymphocyte Response at Study Entry

A total of 131 female patients with HPV16 (type 16) infection was recruited for the study. At the time of enrollment, the IGRA-based HPV16 E7-specific T cell response of PBMCs was assessed and cervical biopsy diagnosis was performed. There were 42 patients without intraepithelial lesions or malignant lesions (NILM), 33 patients with low-grade SIL (LSIL), 39 patients with high-grade SIL (HSIL), and 17 patients with cervical cancer (**Table 1**). Patients in the NILM, LSIL, and HSIL groups were followed for a period of 12 months. Their HPV genotyping results and clinical histological results were also collected at study entry and at the end of the study.

**Table 1:** Clinical characteristics of enrolled patients at study entry (n = 134).

Characteristic	N
Median age	41
Age range	23–67
**Pathological diagnosis of HPV infected**	
NILM	42 (31.3%)
LSIL	33 (24.6%)
HSIL	39 (29.1%)
Cancer	17 (12.7%)
HPV status	
HPV 16 positive	131
HPV negative	3

NILM, negative for intraepithelial lesion or malignancy; LSIL, low-grade squamous intraepithelial lesion; HSIL, high-grade squamous intraepithelial lesion; HPV, human papilloma virus.

We first performed an HPV type16 E7 ROP-based IGRA to assess the status of HPV-related T cell immunity. In the NILM group, 11 patients (26.2%) tested positive for HPV16 E7-specific T cell response and 31 patients (73.8%) had a negative response. In the LSIL group, 14 patients (42.4%) tested positive and 19 patients (57.6%) tested negative for the HPV16-specific T cell response. In the HSIL group, 15 patients (38.5%) were positive and 24 (61.5%) negative for the HPV16 E7 T cell response. All HSIL patients underwent surgery to remove the local pathological lesions. Of the 17 cervical cancer patients, only 1 (5.9%) patient showed a positive HPV16 E7-specific T cell response, and 16 (94.1%) patients presented a negative response (**Table 2**).

**Table 2:** Results of HPV16 E7-specific T lymphocyte response at study entry.

Pathological diagnosis	T+	T−
**NILM (n = 42)**	11 (26.2%)	31 (73.8%)
**LSIL (n = 33)**	14 (42.4%)	19 (57.6%)
**HSIL (n = 39)**	15 (38.5%)	24 (61.5%)
**Cancer (n = 17)**	1 (5.9%)	16 (94.1%)

T+, positive HPV16 E7-specific T lymphocyte response;

T−, negative HPV16 E7-specific T lymphocyte response.

### The Low HPV16-Specific T Cell Response in Peripheral Blood is Relevant to the Persistence of HPV Infection

To determine whether the HPV or HPV peptide-specific T cell response was relevant to the clearance of the persistent HPV infection in different pathogenetic groups, at the end of the one-year follow-up period, HPV genotyping was performed on cervical exfoliated cell samples. In the NILM group, only 2 of 11 patients (18.2%) with a positive HPV16 E7-specific T cell response at the start of the study sustained HPV16 infection. However, 11 of 31 patients (35.5%) who were negative for the HPV16 ROP-specific T cell response harbored a persistent infection. The persistent infection rate was 1.95 times higher than that of patients with positive responses (P = 0.005). In the LSIL group, 2 of 14 patients (14.3%) with a positive HPV16 ROP-specific T cell response sustained a HPV16 infection, while 12 of 19 patients (63.2%) with a negative HPV16-specific T cell response were persistently infected. The persistent infection rate was 4.42 times higher than that of patients with positive response (P <0.0001). In the HSIL group, 39 patients with HPV16 virus infection and high lesions were diagnosed at the time of enrollment, and all these patients underwent surgery. After one year, only 1 of 15 patients (6.7%) with a positive HPV16 specific T cell response sustained HPV16 infection, but 8 of 24 patients (33.3%) with a negative T cell response sustained HPV16 infection. The persistent infection rate was 4.97 times higher than in patients with positive HPV16-specific T cell response (P <0.0001) (**Table 3**). Therefore, the low HPV16 specific T cell response is highly correlated with the persistence of HPV16 infection.

**Table 3:** Results of HPV test from women with HPV16 E7-specific T lymphocyte response at study entry and exit.

		Entry		Exit		P-Value
		HPV positive	HPV negative	HPV positive	HPV negative	
**NILM (n = 42)**	T+	11 (26.2%)	0	2 (18.2%)	9 (81.8%)	0.005
	T−	31 (73.8%)	0	11 (35.5%)	20 (64.5%)	
**LSIL (n = 33)**	T+	14 (42.4%)	0	2 (14.3%)	12 (85.7%)	<0.0001
	T−	19 (57.6%)	0	12 (63.2%)	7 (36.8%)	
**HSIL (n = 39)**	T+	15 (38.5%)	0	1 (6.7%)	14 (93.3%)	<0.0001
	T−	24 (61.5%)	0	8 (33.3%)	16 (66.7%)	

### The HPV16 E7 ROP-Specific T Cell Response is Associated With Histological Regression

**Figure 2:**
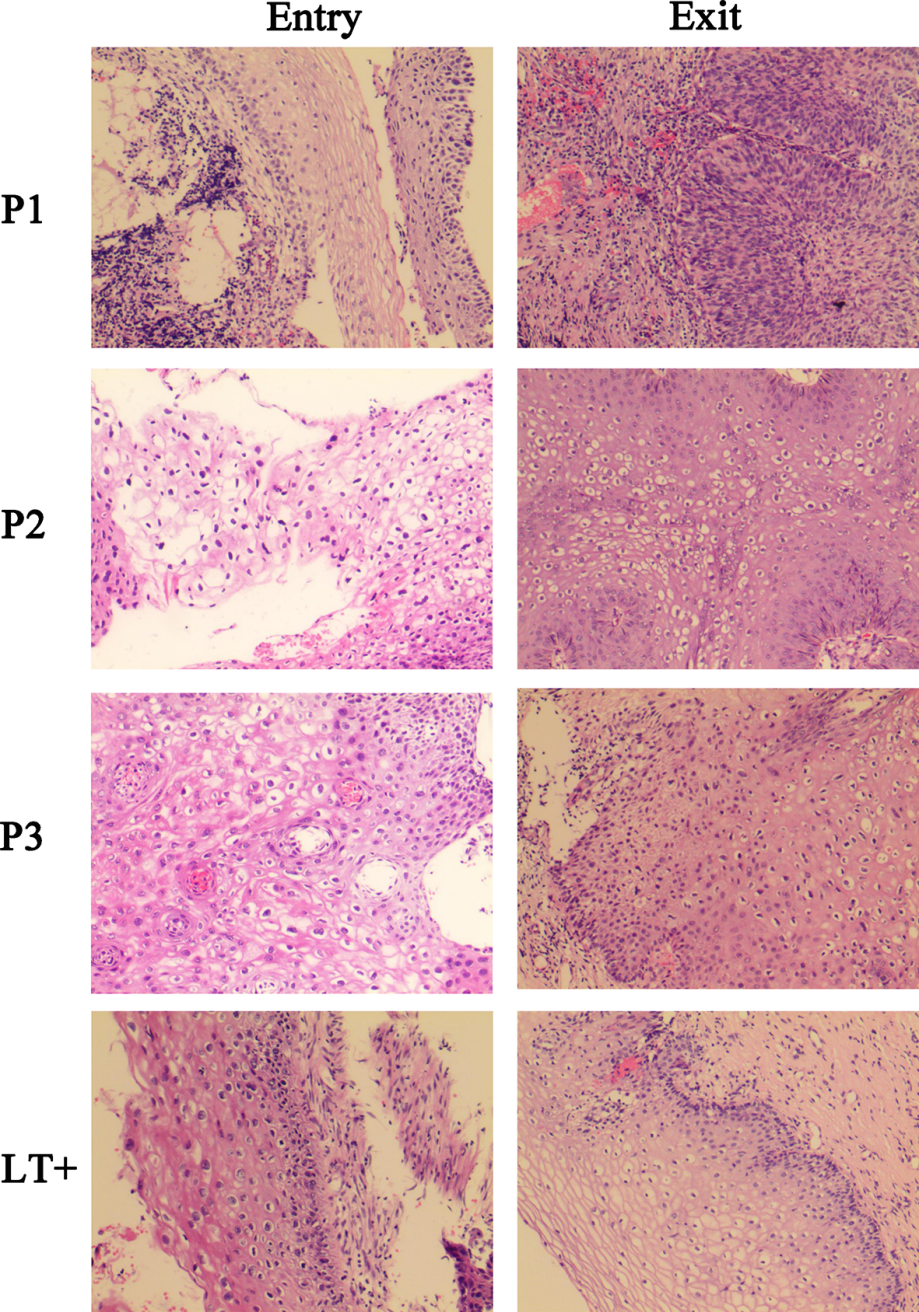
Histological images of three progressed patients and a regressed representative patient. P1, P2, P3, were HPV16-infected patients in NILM cohorts at the study entry. At study exit, P1 progressed to HSIL, and P2, P3 progressed to LSIL. LT+, is a regressed representative of patients positive for the HPV16 E7 specific T lymphocyte response, who was LSIL at study entry and completely regressed at study exit.

To study the influence of the T-cell immunity response on HPV pathogenesis, at the end of this study, a cervical biopsy was performed. The results showed that in all three groups (NILM, LSIL, HSIL), none of the patients with positive specific T cell response against HPV16 E7 presented any further disease deterioration (**Table 4**). Specifically, in the NILM group, 25.8% of patients with negative T cell response retained the same lesion features by pathohistological examination, which was 1.42-fold higher than that of patients who were T cell positive (18.2%) against HPV ROP. The histological regression rate among patients with positive T cells for HPV16 E7 ROP was 81.7%, which was 1.26-fold higher than that of the negative T cell response (64.5%). Three of 31 patients (9.7%) with negative specific T cell response against HPV16 E7 ROP progressed pathologically (P = 0.001); the histological results are shown in **Figure 2**. Among the patients in the LSIL group (n = 33), 31.6% of patients with a negative T cell response to HPV16 E7 ROP maintained the same lesion characteristics on pathohistological examination, which was 2.21-fold higher than that of patients with a positive T cell response (14.3%). The regression rate in T cell negative patients (68.4%) was less (0.8-fold) than those with a positive T cell response (85.7%) (P = 0.002). In the HSIL group (n = 39), at the end of the 12-month study, the 15 patients with positive specific T cell response against HPV16 E7 ROP are in stable condition without histologically observed recurrence (100% regressed); although 1 of the 24 patients with a negative response experienced disease recurrence (P = 0.043) (**Table 4**). Of note, all patients in the HSIL group received surgery before the study had started. This could have contributed to the higher regression rate in this group.

**Table 4:** Results of cervical biopsy test from women with HPV16 E7-specific T lymphocyte response at study exit.

			Cervical biopsies			P-value
			Progressors	Persistors	Regressors	
**NILM (n = 42)**	T+	11 (26.2%)	0	2 (18.2%)	9 (81.7%)	0.001
	T−	31 (73.8%)	3 (9.7%)	8 (25.8%)	20 (64.5%)	
**LSIL (n = 33)**	T+	14 (42.4%)	0	2 (14.3%)	12 (85.7%)	0.002
	T−	19 (57.6%)	0	6 (31.6%)	13 (68.4%)	
**HSIL (n = 39)**	T+	15 (38.5%)	0	0	15 (100%)	0.043
	T−	24 (61.5%)	0	1 (4.2%)	23 (95.8%)	

The NILM, LSIL and HSIL cohorts were classified as Progressors, Persistors or Regressors after 1-year observation.

### The HPV16 E7 ROP-Specific T Cell Response is Relevant for theClinical Outcome

A total of 114 women completed the 12-month study. At the beginning of their hospital visit, all were positive for HPV16 infection by PCR test. Forty patients (35.1%) were positive for the HPV16 E7 ROP-specific T cell response. The remaining 74 patients (64.9%) were negative for the T cell response (**Table 5**). At the most recent follow-up visit, 78 of 114 patients (68.4%) had cleared the HPV16 infection, 3 patients (2.6%) had persistent HPV16 infection and progressed histologically, the remaining 33 patients (28.9%) had persistent HPV16 infection. All 3 progressed patients were negative for the HPV16 E7 ROP-specific T cell response. Only 5/33 (15.2%) HPV16 persistently infected patients had a positive HPV16 E7 specific T cell response, while 28/33 (84.8%) had a negative response. Among patients who were positive for the HPV16 E7 ROP-specific T cell response (n = 40), 87.5% of the patients cleared HPV16 infection, 12.5% had persistent infection and none of them progressed after 12 months. While in patients with a negative HPV16 E7 ROP-specific T cell response (n = 74), 43 of 74 (58.1%) patients had cleared HPV16 infection, 31 of 74 (41.9%) persistently infected, and 3 of persistently infected patients histologically progressed (**Table 5**).

**Table 5:** Clinical outcomes of patients with HPV16 E7-specific T cell response.

Entry	Exit			P-value	
HPV16 Positive (n = 114)	Clearance (n = 78)	Persistence (n = 33)	Progression (n = 3)	
**T+ (n = 40, 35.1%)**	35 (87.5%)	5 (12.5%)	0	<0.0001
**T**− **(n = 74, 64.9%)**	43 (58.1%)	28 (37.8%)	3 (4.1%)

**Figure 3:**
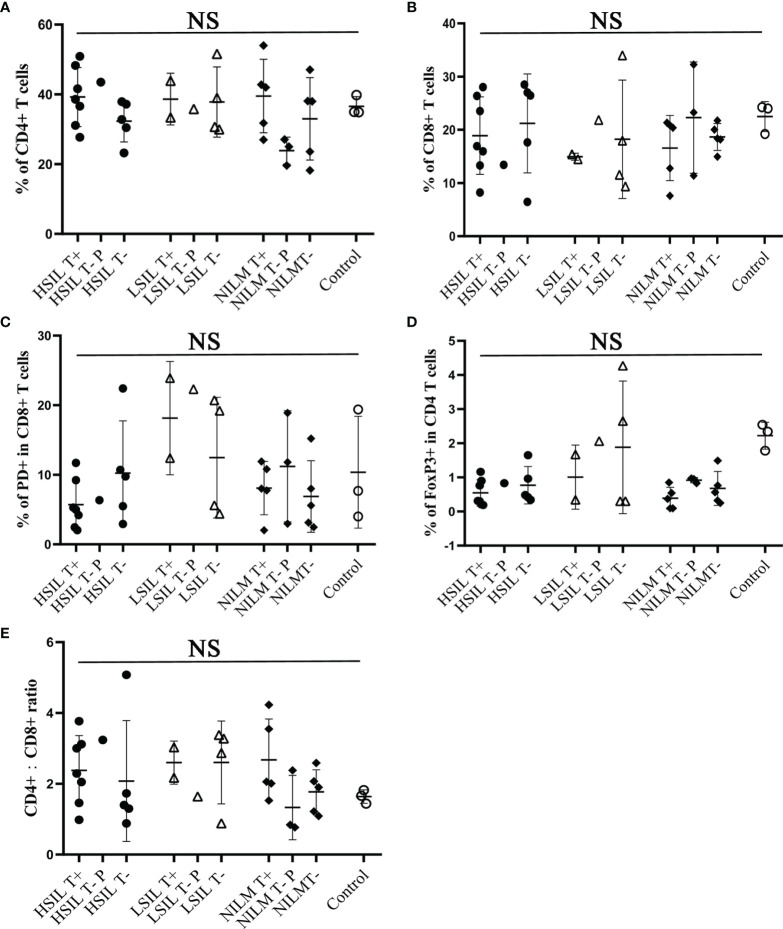
Flow cytometric analysis of CD8, CD4, CD4/CD8 ratio, PD-1 and FoxP3 from peripheral blood CD3^+^ T cells of HPV 16 infected cohorts. In total 32 patients were analyzed. **(A, B)** Frequency of CD8 or CD4 expression on CD3^+^ T cells. **(C)** Frequency of PD-1 expression on CD8^+^/CD3^+^ T cells. **(D)** Frequency of FoxP3 expression on CD4^+^/CD3^+^ T cells. **(E)** CD4:CD8 ratio. T+, sample with positive HPV16-specific T cell response; T−, sample with negative HPV16-specific T cell response; P, persistent HPV16 infection; Control, samples from 3 women with no history of HPV16 infection. NS, no significance.

We also performed flow cytometry using PBMCs of 32 patients and 3 non-infected controls, detecting the proportion of CD8^+^ T cells, CD4^+^ T cells, CD4^+^:CD8^+^ ratio, CD8^+^ T cells expressing PD-1 and regulatory T cells expressing Foxp3. The results showed no correlation with prognosis (**Figure 3**). The detailed gating information of flow cytometry data can be found in **Supplementary Figures 1** and **2**.

## Discussion

The purpose of this study was to evaluate the role of HPV specific T cell immunity in the clinical outcome of HPV infected patients. We used ROP-HPV E7, LRMK-linked overlapping peptides covering the HPV E7 sequence (12) as the antigenic agent to test the specific T cell activity of the patient. This study evaluated cellular immunity specific to HPV16 E7 in 131 women with HPV16 infection. Only one of 17 (5.9%) patients with cervical cancer had a positive HPV16 E7-specific T cell response, dramatically lower than the precancer patient groups (26.5% in NILM, 42.4% in LSIL, and 38.5% in HSIL). Our data are consistent with others who have shown that immunosuppressed humans or animals have increased risks of HPV infection and associated dysplasia (13, 14), suggesting that immune reactivity is associated with virus elimination and disease clearance.

The progression from HPV infection to malignant tumor requires viral escape from host immunity (15). With a properly functioning immune system, up to 80–90% of HPV infection was cleared in two years (16). This has raised a dilemma in the management of HPV infection, that is, whether costly, complicated, and invasive medical surgical intervention should be provided to an HPV infected patient once diagnosed as HPV-positive, or should this wait until the cancer is developed? A valid prognostic method is an unmet need and will be helpful for clinicians and patients for clinical decision-making. Most current clinically available diagnostic methods focus on either detecting the presence of virus by PCR or detecting tumor presence by histological examination. There is no good prognosis method available to monitor HPV-associated human immune responses. Consequently, patients may either undergo unnecessary surgery if the decision is made based only on PCR findings or patients may receive intervention when it is too late to achieve effective treatment. Unfortunately, in many cases no action has been taken because clinicians take for granted that most HPV infections resolved automatically. Our results support that the monitoring of T cell-based immunity may be a suitable approach for prognostic purposes. The results of this pilot study suggest that if good T cell immunity is detected, then it is likely that HPV infection will be cleared. In the absence of T cell immunity, interventions should be taken to stop the malignant progression.

The WHO proposes 3 tiers of management for HPV related diseases. Tier 1: prophylactic vaccines for individuals without HPV infection; Tier 2: therapy for HPV-infected individuals who have not progressed to malignancy; Tier 3: therapies for HPV-infected individuals who have progressed to a late malignant stage. The findings from this study are relevant to the development of T cell-based therapeutic vaccines for Tier 2 and 3 therapies. Although prophylactic vaccines against HPV are available, these vaccines are not effective for those who have already been infected with HPV (17). The fact that T cell immunity is important for resolving HPV infection indicates that a T cell-based vaccine strategy will be feasible and beneficial to HPV-infected individuals having low immunity. Indeed, there are several promising clinical trials evaluating the role of enhanced T cell-based immunity (4). For example, synthetic long overlapping peptides (SLP) derived from HPV E6 and E7 are in a Phase II clinical trial in HPV-infected high-grade vulvar intraepithelial neoplasia have shown an 80% partial effect response and 47% complete regression (18). The combination of SLP with anti-PD-1 antibody has shown a achieve of a synergetic effect in incurable HPV 16-related cancer (19). Together with these promising trials, our data support the hypothesis that T-cell-based immunity plays an instrumental role in the containment of HPV infection.

## Methods

### Patients

Women with HPV16 infection aged 23 to 67 years attending routine cervical cancer screening at the Changzhou Maternity and Child Health Care Hospital affiliated with Nanjing Medical University were asked to enroll in the study after giving their written informed consent. The study was approved by the Internal Review Board. Exclusion criteria were unwillingness or unavailability to follow up 12 months after recruitment. The patients enrolled were not pregnant and had no prior medical history of immune disorders. At the beginning of the study, a cervico-vaginal sample was collected from each participating subject for cytological testing and HPV DNA genotyping. Women positive for HPV 16 genotyping were recruited and referred for colposcopy and biopsy. Blood samples from enrolled patients were collected for the ELISPOT test. Patients would be recalled every 6 months. At the end of the study, 114 HPV 16 positively infected patients completed the 12-month follow-up study.

### Histology

HPV16-positive patients were referred to colposcopy biopsy. Cervical or vaginal wall biopsy tissues were formalin-fixed, paraffin-embedded, and stored at room temperature. The tissue size was at least 3 mm for manual slicing with a rotary slicer. Serial sections were cut and stained with hematoxylin and eosin (HE). All images used in this study were acquired using an Olympus BX45 microscope equipped with 20× objective (Olympus, America). All histological sections were reviewed by a single histopathologist for the purpose of this study, who was blinded to other clinical information. The cases were classified as NILM, LSIL, HSIL, or cancer.

### Mixed HPV16 E7 Peptides and ROP-HPV16 E7

Four synthetic overlapping peptides covering the entire sequence of the HPV16 E7 protein were synthesized (Ontores Biotech, China): Peptide 1, I R T L E D L L M G T L G I V C P I C S Q K P; peptide 2, M H G D T P T L H E Y M L D L Q P E T T D L Y C Y E Q L N D S S E E E; peptide 3, E Q L N D S S E E E D E I D G P A G Q A E P D R A H Y N I V T F C C K; and peptide 4, H Y N I V T F C C K C D S T L R L C V Q S T H V D I R T L E D L L M G.

The ROP-HPV16 E7 recombinant overlapping peptide is an artificial protein that contains E7 overlapping peptides linked to LRMK (a cathepsin S cleavage site). ROP-HPV16 E7 were expressed and purified as described elsewhere (12). Briefly, BL21 transformed with ROP-HPV16 E7 (DE3) was cultured in LB broth (50 µg/ml Kanamycin) overnight at 37°C and was then diluted with fresh LB broth and cultured until the OD600 reached 0.6. Cells were harvested 16 h after induction with IPTG (0.5 mM). Cell pellets were resuspended in lysis buffer (25 mM TrisHCl, 200 mM NaCl, 2% Triton X-100, 10 mM imidazole, pH8.0) and lysed by sonication. Soluble fractions were collected. The Ni-NTA resin was then added to the soluble fractions for 30 min, followed by washing with 30 resin volumes of lysis buffer and eluted with lysis buffer containing 200 mM imidazole. The eluted protein was dialyzed at 4°C in PBS buffer containing 10% glycerol.

### Isolation of Human Peripheral Blood Mononuclear Cells

Heparin-treated human blood (10 ml) from patients was carefully added to the lymphocyte-separation medium (density = 1.077) and centrifuged at 1,500×*g* for 25 min. The PBMC layer was transferred to a new tube and was washed twice with RPMI 1640 medium.

### Enzyme-Linked Immunospot Assay

The assays were performed using ELISPOT kits (Mabtech, Sweden). PBMCs (5 × 10^5^ cells/well) were stimulated overnight with 2 µM ROP-HPV16 E7 or with mixed E7 peptides in anti-IFN-γ-Ab precoated plates (Millipore, Bedford, MA). Cells were discarded and biotinylated anti-IFNγ antibodies were added for 2 h at room temperature, followed by an additional 1 h incubation at room temperature with enzyme-labeled strepavidin. After the color developed, the reaction was stopped by washing the plates with tap water, and the plates were air dried. Spots were counted with an Elispot reader (Autoimmun Diagnostike, Strasburg, Germany).

### Flow Cytometry Analysis

PBMCs were washed once with 1 ml ice-cold FACS buffer (2% FCS in PBS) and the cell density was adjusted to 1 × 10^6^ cells/100 μl. APC-conjugated anti-human CD3 monoclonal antibody (BD, USA), FITC-conjugated anti-human CD8 (BD, USA), PE-conjugated anti-human CD279 monoclonal antibody (BD, USA) and FVS620 (BD, USA) were added and incubated in the dark for 15 min at room temperature. For FoxP3 staining, PBMCs were first stained with FITC-conjugated anti-Human CD4 monoclonal antibody (BD, USA), APC-conjugated anti-Human CD25 monoclonal antibody (BD, USA) and FVS620 (BD, USA) for 15 min in the dark at room temperature. After fixation and permeabilization, the cells were stained with PE-conjugated anti-human FoxP3 monoclonal antibody (BD, USA) overnight at 4°C. The cells were then washed three times with 1 ml of FACS buffer, resuspended in 0.5 ml of FACS fixing buffer (BD, USA) and acquired using CytoFLEX S flow cytometer (Beckman, USA). The data was analyzed by FlowJo software version VX (ThreeStar, San Carlos, CA, USA).

### Statistical Analysis

We used the *t*-test for continuous variables and Chi-square test for categorical variables. A P-value <0.05 in the two-sided test was considered statistically significant. Statistical analyses were performed using IBM SPSS Statistics version 25 (SPSS, Chicago, USA). **Figures 1** and **3** were generated and analyzed using GraphPad Prism version 8 (GraphPad Software Inc.).

## Data Availability Statement

The original contributions presented in the study are included in the article/**Supplementary Material**. Further inquiries can be directed to the corresponding authors.

## Ethics Statement

The studies involving human participants were reviewed and approved by the Ethics Committee of Changzhou Maternal and Child Health Hospital. The patients/participants provided their written informed consent to participate in this study.

## Author Contributions

SJ and WL designed and wrote the manuscript. LZ collected patient samples and performed most of the experiments. XinS and WL carried out ELISPOT analysis. QZ performed flow cytometry assays. ZM and XiaS helped to collect patient samples. ZM and AJ participated in the statistical analysis; RZ and JZ participated in the experimental design and project management. All authors contributed to the article and approved the submitted version.

## Funding

This research was supported by grants from the general scientific research project of Jiangsu Provincial Health Commission (H2018018).

## Conflict of Interest

Authors XinS, QZ, AJ and RZ were employed by company Oxford Vacmedix Co. Ltd. Author WL was employed by company Oxford Vacmedix Co. Ltd. and Shanghai JW Inflinhix Co. Ltd.

The remaining authors declare that the research was conducted in the absence of any commercial or financial relationships that could be construed as a potential conflict of interest.

## Publisher’s Note

All claims expressed in this article are solely those of the authors and do not necessarily represent those of their affiliated organizations, or those of the publisher, the editors and the reviewers. Any product that may be evaluated in this article, or claim that may be made by its manufacturer, is not guaranteed or endorsed by the publisher.

## References

[1] Hu Z Ma D. The Precision Prevention and Therapy of HPV-Related Cervical Cancer: New Concepts and Clinical Implications. Cancer Med (2018) 7:5217–36. doi: 10.1002/cam4.1501 PMC6198240 30589505

[2] Morrow MP Kraynyak KA Sylvester AJ Dallas M Knoblock D Boyer JD. Clinical and Immunologic Biomarkers for Histologic Regression of High-Grade Cervical Dysplasia and Clearance of HPV16 and HPV18 After Immunotherapy. Clin Cancer Res (2018) 24:276–94. doi: 10.1158/1078-0432.CCR-17-2335 PMC6956401 29084917

[3] Choi YJ Park JS. Clinical Significance of Human Papillomavirus Genotyping. J Gynecol Oncol (2016) 27:e21. doi: 10.3802/jgo.2016.27.e21 26768784 PMC4717226

[4] Lee SJ Yang A Wu TC Hung CF. Immunotherapy for Human Papillomavirus-Associated Disease and Cervical Cancer: Review of Clinical and Translational Research. J Gynecol Oncol (2016) 27:e51. doi: 10.3802/jgo.2016.27.e51 27329199 PMC4944018

[5] Josefsson AM Magnusson PK Ylitalo N Sorensen P Qwarforth-Tubbin P Andersen PK. Viral Load of Human Papilloma Virus 16 as a Detetmination for Development of Cervical Carcinoma Insitu:a Nested Case Control Study. Lancet (2000) 355:2189–93. doi: 10.1016/S0140-6736(00)02401-6 10881891

[6] Xie JY Xie JN Yu KJ Dong GQ Cai CL Xu GH. The Relationship Between the Load and Duration of High-Risk Human Papillomavirus and Cervical Lesion in Pregnant Women. Chin J Clin Obstet Gynecol (2015) 16:550–2. doi: 10.13390/j.issn.1672-1861.2015.06.022

[7] Shalapour S Karin M. Immunity, Inflammation, and Cancer: An Eternal Fight Between Good and Evil. J Clin Invest (2015) 125:3347–55. doi: 10.1172/JCI80007 PMC4588298 26325032

[8] Sarkara AK Tortolero-Luna G Follen M Sastry KJ. Inverse Correlation of Cellular Immune Responses Specific to Synthetic Peptides From the E6 and E7 Oncoproteins of HPV-16 With Recurrence of Cervical Intraepithelial Neoplasia in a Cross-Sectional Study. Gynecol Oncol (2005) 99:S251–61. doi: 10.1016/j.ygyno.2005.07.099 16188303

[9] Nakagawa M Stites DP Palefsky JM Kneass Z Moscicki AB. CD4-Positive and CD8-Positive Cytotoxic T Lymphocytes Contribute to Human Papillomavirus Type 16 E6 and E7 Responses. Clin Diagn Lab Immunol (1999) 6:494–8. doi: 10.1128/CDLI.6.4.494-498.1999 PMC95714 10391849

[10] Nakagawa M Stites DP Patel S Farhat S Scott M Hills NK. Persistence of Human Papillomavirus Type 16 Infection is Associated With Lack of Cytotoxic T Lymphocyte Response to the E6 Antigens. J Infect Dis (2000) 182:595–8. doi: 10.1086/315706 10915094

[11] Mirabello L Yeager M Yu K Clifford GM Xiao Y Zhu B. HPV16 E7 Genetic Conservation Is Critical to Carcinogenesis. Cell (2017) 170:1164–74. doi: 10.1016/j.cell.2017.08.001 PMC5674785 28886384

[12] Cai L Zhang J Zhu R Shi W Xia X Edwards M. Protective Cellular Immunity Generated by Cross-Presenting Recombinant Overlapping Peptide Proteins. Oncotarget (2017) 8:76516–24. doi: 10.18632/oncotarget.20407 PMC5652724 29100330

[13] Ahdieh L Muñoz A Vlahov D Trimble CL Timpson LA Shah K. Cervical Neoplasia and Repeated Positivity of Human Papillomavirus Infection in Human Immunodeficiency Virus-Seropositive and -Seronegative Women. Am J Epidemiol (2000) 151:1148–57. doi: 10.1093/oxfordjournals.aje.a010165 10905527

[14] Jain S Moore RA Anderson DM Gough GW Stanley MA. Cell-Mediated Immune Responses to COPV Early Proteins. Virology (2006) 356:23–34. doi: 10.1016/j.virol.2006.07.032 16949120

[15] Smola S. Immunopathogenesis of HPV-Associated Cancers and Prospects for Immunotherapy. Viruses (2017) 9:254. doi: 10.3390/v9090254 PMC5618020 28895886

[16] Moscicki AB Shiboski S Hills NK Powell KJ Jay N Hanson EN. Regression of Low-Grade Squamous Intra-Epithelial Lesions in Young Women. Lancet (2004) 364:1678–83. doi: 10.1016/S0140-6736(04)17354-6 15530628

[17] Hildesheim A Herrero R Wacholder S Rodriguez AC Solomon D Bratti MC. Effect of Human Papillomavirus 16/18 L1 Viruslike Particle Vaccine Among Young Women With Preexisting Infection: A Randomized Trial. JAMA (2007) 298:743–53. doi: 10.1001/jama.298.7.743 17699008

[18] Kenter GG Welters MJ Valentijn AR Lowik MJ Berends-van der Meer DM Vloon AP. Vaccination Against HPV-16 Oncoproteins for Vulvar Intraepithelial Neoplasia. N Engl J Med (2009) 361:1838–47. doi: 10.1056/NEJMoa0810097 19890126

[19] Massarelli E William W Johnson F Kies M Ferrarotto R Guo M. Combining Immune Checkpoint Blockade and Tumor-Specific Vaccine for Patients With Incurable Human Papillomavirus 16–Related Cancer A Phase 2 Clinical Trial. JAMA Oncol (2019) 5:67–73. doi: 10.1001/jamaoncol.2018.4051 30267032 PMC6439768

