# Supplementary figures and images for: HPV-16 E7-Specific Cellular Immune Response in Women With Cervical Intraepithelial Lesion Contributes to Viral Clearance: A Cross-Sectional and Longitudinal Clinical Study
Source: Front Immunol. 2022 Jan 13;12:768144. doi: 10.3389/fimmu.2021.768144 (PMC8793279; doi:10.3389/fimmu.2021.768144)

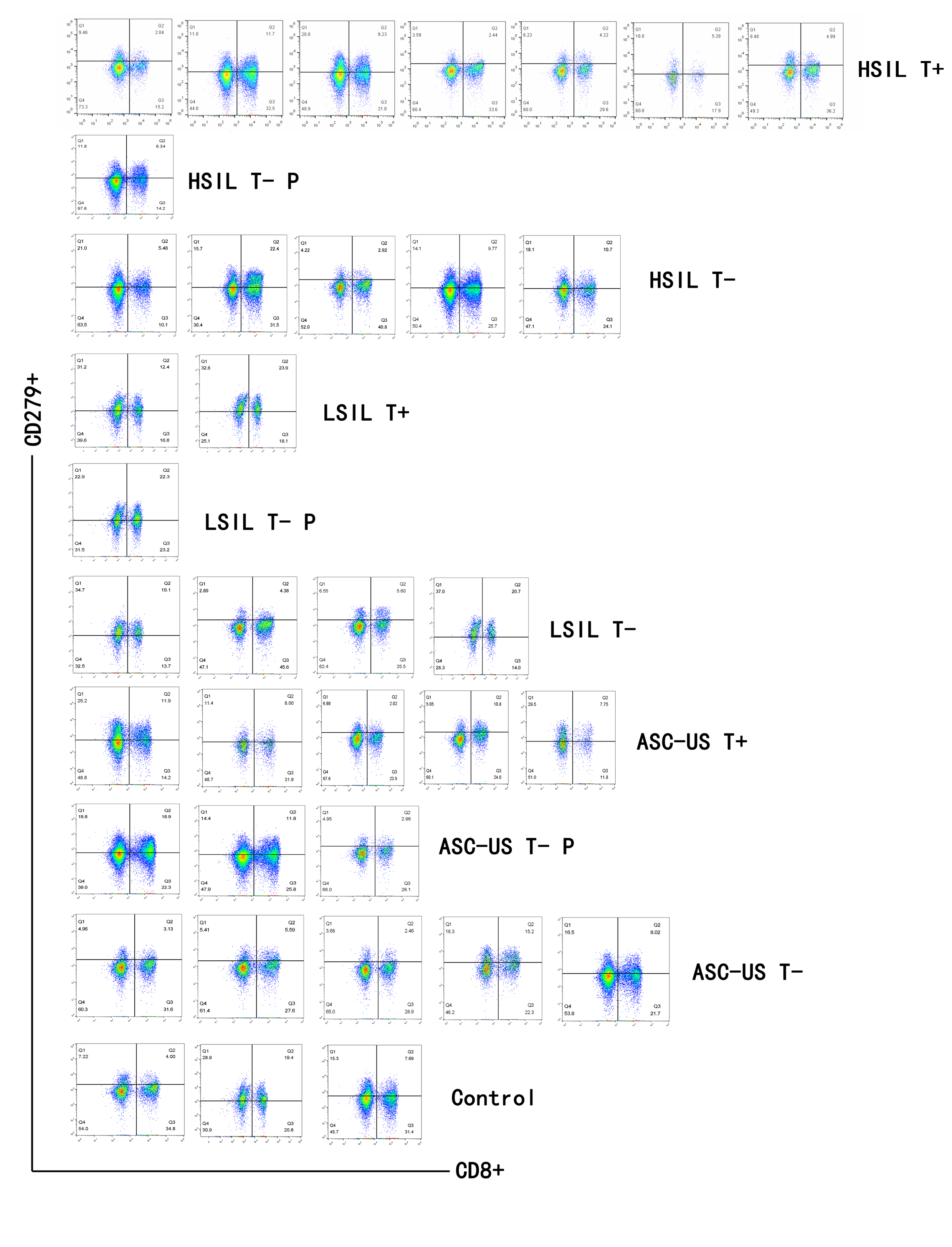

Supplement: Supplementary file 1 [file Image_1.tif]

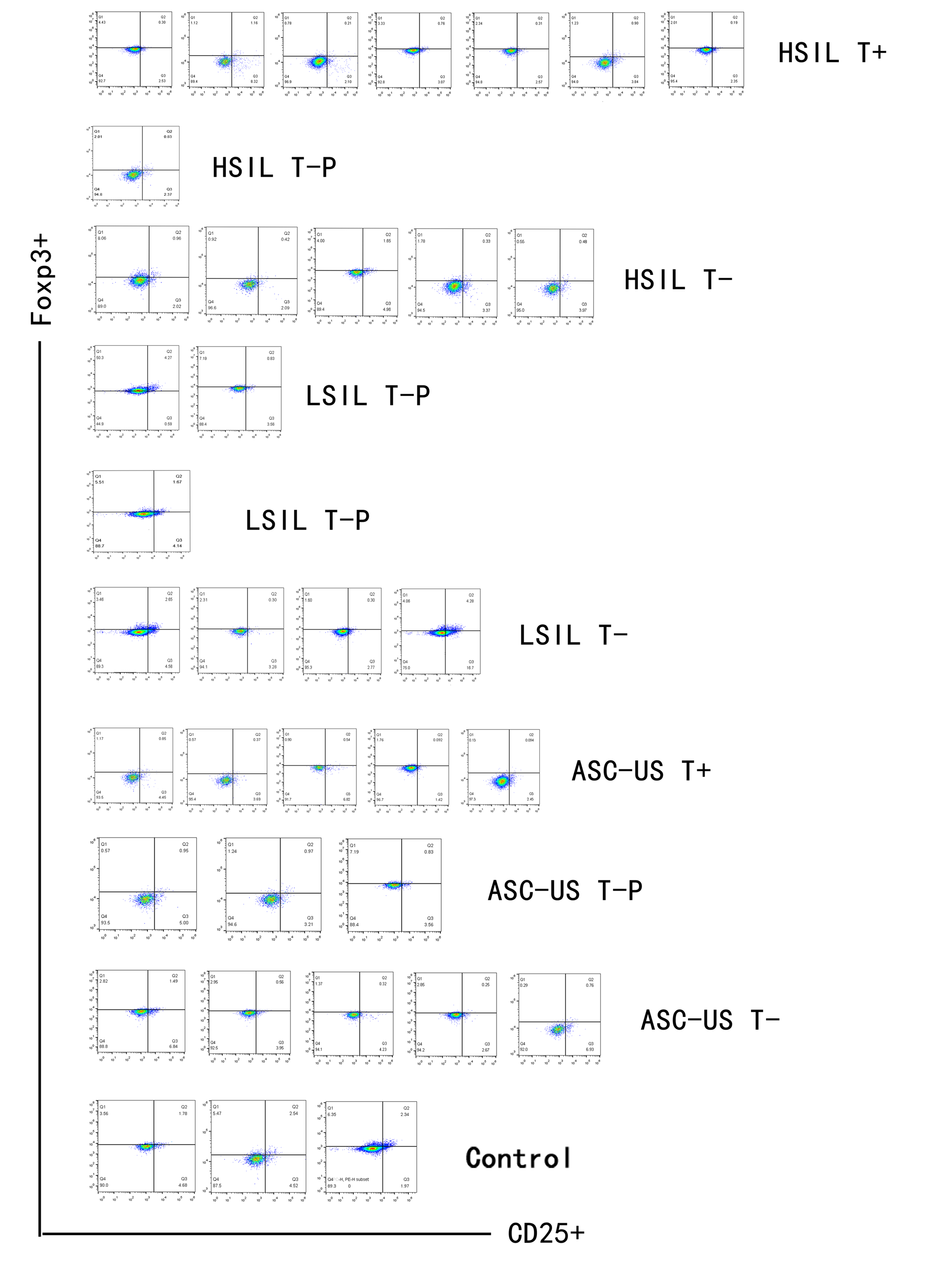

Supplement: Supplementary file 2 [file Image_2.tif]
